# Assessing clinical efficacy of polyp detection models using open-access datasets

**DOI:** 10.3389/fonc.2024.1422942

**Published:** 2024-08-01

**Authors:** Gabriel Marchese Aizenman, Pietro Salvagnini, Andrea Cherubini, Carlo Biffi

**Affiliations:** ^1^ Cosmo Intelligent Medical Devices, Dublin, Ireland; ^2^ Milan Center for Neuroscience, University of Milano–Bicocca, Milano, Italy

**Keywords:** artificial intelligence, deep learning, polyp detection, colonoscopy, FROC

## Abstract

**Background:**

Ensuring accurate polyp detection during colonoscopy is essential for preventing colorectal cancer (CRC). Recent advances in deep learning-based computer-aided detection (CADe) systems have shown promise in enhancing endoscopists’ performances. Effective CADe systems must achieve high polyp detection rates from the initial seconds of polyp appearance while maintaining low false positive (FP) detection rates throughout the procedure.

**Method:**

We integrated four open-access datasets into a unified platform containing over 340,000 images from various centers, including 380 annotated polyps, with distinct data splits for comprehensive model development and benchmarking. The REAL-Colon dataset, comprising 60 full-procedure colonoscopy videos from six centers, is used as the fifth dataset of the platform to simulate clinical conditions for model evaluation on unseen center data. Performance assessment includes traditional object detection metrics and new metrics that better meet clinical needs. Specifically, by defining detection events as sequences of consecutive detections, we compute per-polyp recall at early detection stages and average per-patient FPs, enabling the generation of Free-Response Receiver Operating Characteristic (FROC) curves.

**Results:**

Using YOLOv7, we trained and tested several models across the proposed data splits, showcasing the robustness of our open-access platform for CADe system development and benchmarking. The introduction of new metrics allows for the optimization of CADe operational parameters based on clinically relevant criteria, such as per-patient FPs and early polyp detection. Our findings also reveal that omitting full-procedure videos leads to non-realistic assessments and that detecting small polyp bounding boxes poses the greatest challenge.

**Conclusion:**

This study demonstrates how newly available open-access data supports ongoing research progress in environments that closely mimic clinical settings. The introduced metrics and FROC curves illustrate CADe clinical efficacy and can aid in tuning CADe hyperparameters.

## 1 Introduction

CRC originates from adenomas or serrated polyps, which can progress to cancer over time. CRC ranks third in cancer-related deaths and second in mortality worldwide (1, 2). Colonoscopy, recognized as the gold-standard method for diagnosing and preventing CRC, owes its effectiveness primarily to its capability to detect and then remove these polyps. However, endoscopists’ proficiency and vigilance significantly influence polyp detection (3). Population-based studies have shown miss rates of up to 26% for adenomas and 27% for serrated polyps, with missed polyps accounting for 57.8% of interval CRCs (4, 5). Numerous randomized trials have demonstrated the substantial benefits of CADe-assisted colonoscopy compared to standard practices, consistently showing higher adenoma detection rates (ADR). These rates are elevated regardless of polyp size, location, and morphology, as confirmed by several meta-analyses (6, 7). This advancement promises not only to improve diagnostic performance but also to ensure more uniform screening outcomes (8–11).

The primary challenge for CADe systems is to achieve high polyp detection rates (recall), typically assessed in clinical literature as the proportion of polyps detected in at least one frame (12), while also maintaining a low rate of FPs, which are commonly reported as the average number per patient throughout an entire video (13). The occurrence of FPs, often triggered by polyp-like structures and artifacts within the complex colon environment, can prolong examination times and increase endoscopists’ workload and fatigue. Generally, efforts to reduce FP alerts may compromise detection accuracy, especially at the initial moment of polyp discovery when polyps are often poorly framed or characterized by small bounding boxes (14, 15). Increasing sensitivity to detect these small or ambiguously shaped polyps raises the risk that the polyp detection algorithm may be deceived by similar non-polyp structures (16, 17). Additionally, CADe systems for polyp detection must operate in real-time, constraining the use of larger, more computationally demanding learning-based architectures and necessitating the use of images at a downsampled resolution, which ultimately may reduce detection accuracy.

The definition of CADe FPs varies widely in clinical literature, complicating the assessment of clinical utility and comparison across trials (12, 14, 18). Recent studies suggest that over 95% of per-frame FPs are disregarded by endoscopists without affecting withdrawal time, indicating that consecutive detection events lasting 1 or 2 seconds could be a more effective measure of FPs per video (19). Furthermore, beyond mere detection, the true value of CADe systems lies in their ability to quickly identify polyps as they first appear, facilitating early alerts for endoscopists and reducing the likelihood of missed diagnoses. This underscores the importance of developing metrics that capture CADe system performance during these critical moments of detection and, more broadly, that better align with clinical needs (3, 20).

Open-access datasets have significantly contributed to the research and development of deep learning-based models for polyp detection (17, 21–24). This process typically involves developing these models and fine-tuning their hyperparameters using traditional computer vision metrics such as precision and recall, computed at the bounding box or frame level (24). However, these metrics often fail to accurately inform about the models’ performance in clinical settings, where assessments are usually conducted at the polyp or video/patient level. This introduction of more nuanced metrics for whole-procedure evaluations, such as per-polyp recall and per-patient FP rates as previously discussed, is emphasized in **Table 1**. In this direction, the recent introduction of the REAL-Colon dataset (17), consisting of 60 annotated whole-procedure videos from multiple centers, provides a new opportunity to extend evaluations beyond traditional per-bounding box and per-frame assessments.

**Table 1:** The clinical deployment of deep polyp detection algorithms requires additional data, metrics, and parameters to accurately quantify the model’s clinical efficacy and fine-tune its operational parameters.

	Detection Algorithm	Deployed Application
**Data**	Video Frames	Whole-Procedure Video
**Metrics**	Recall Per-Box/Per-Frame	Recall Per-Polyp
	Precision Per-Box/Per-Frame	False Positives Per-Patient
**Parameters**	Model Hyperparameters	Event Duration *τ*
	(Detection Threshold *δ*, etc.)	Detection Window *σ*

In this study, we consolidate five open-access polyp detection datasets into a unified, multi-center database, establishing data splits for model training and validation. We introduce clinically relevant metrics—such as per-polyp recall at early detection stages and per-patient FP rates—that complement traditional polyp detection metrics, thereby enhancing the characterization of model clinical behavior. Our primary goal is to develop an open-access platform that encompasses both data and metrics for CADe system development and benchmarking under conditions that closely mimic clinical environments. To this end, we leverage full-procedure multi-center videos from the REAL-Colon dataset as the testing component of this platform, which aids in assessing the generalization of CADe systems across unseen centers. Our secondary aim is to demonstrate the clinical relevance of our newly proposed metrics by refining and evaluating the clinical behavior of CADe systems. Through the state-of-the-art YOLOv7 detection model, chosen for its real-time effectiveness, ease of use, and widespread adoption within the polyp detection and broader object detection communities (20, 25), we aim at showcasing how this platform supports clinical efficacy evaluations of CADe systems and their refinement.

## 2 Materials and methods

### 2.1 Datasets

For this study, we assembled a collection of five open-access datasets, SUN, KUMC, LDPolypVideo, PolypGen and REAL-Colon databases. A comprehensive summary of each dataset is provided in **Table 2**, detailing dataset contributions to this research in terms of frames (positives and negatives) and distinct polyps. Each database includes both frames depicting polyps annotated with bounding boxes (positive frames), as well as additional frames depicting non-polyp scenes (negative frames). The SUN database, collected at Showa University Northern Yokohama Hospital, comprises 158,690 frames including 100 polyps (23). The KUMC dataset comprises 80 polyp video-clips sourced from the University of Kansas Medical Center, totaling 37,899 frames (21). The LDPolypVideo dataset contains 40,266 frames and an additional 103 videos with unannotated frames. From these unannotated videos, we extracted for this work every fifth frame using ffmpeg software to add 99,374 additional negative frames (22). The PolypGen dataset, contributed by six centers across Europe and Africa, offers a compilation of 8,037 frames, encompassing various endoscopic systems and expert evaluations (24). The REAL-Colon dataset comprises 60 annotated videos, each originating from one of four distinct video acquisition cohorts across six centers (cohort 001 - three centers in the United States, 002 - Italy, 003 - Austria, 004 - Japan), utilizing various endoscopes, and totaling 2,757,723 frames (17). Participants included in the dataset are all aged 40 or older, undergoing colonoscopy for primary CRC screening, post-polypectomy surveillance, positive fecal immunochemical tests, or diagnosis based on symptoms/signs. The exclusion criteria for this dataset include a history of CRC or inflammatory bowel disease, previous colonic resection, emergency colonoscopy procedures, or ongoing antithrombotic therapy. Notably, each video in this dataset captures an entire colonoscopy procedure from start to finish at maximum resolution, without any pauses or interruptions, closely mimicking the actual clinical environment in which CADe systems are used.

**Table 2:** Summary of open-access datasets used for polyp detection in this study, detailing frame counts, resolution, and distribution between positive (polyp) and negative (non-polyp) frames.

Dataset	Frames	Positive	Negative	Polyps	Resolution
KUMC (21)	37,899	35,981	1,918	80	various
SUN (23)	158,690	49,136	109,554	100	1240×1080
LDPolypVideo (22)	139,640	33,875	105,765	200	560×480
PolypGen (24)	8,037	3,121	4,916	unclear	various
REAL-Colon (17)	2,757,723	342,109	2,415,614	131	1920×1080

### 2.2 Experimental setup

Using the available data, we have constructed six different training sets as reported in **Table 3**. We combined KUMC, SUN, and LDPolypVideo (including both the annotated and additional negative frames sampled) to create a training dataset named the Unified Colonoscopy Dataset (UCD). To this dataset, either the first five videos from each of the four dataset cohorts (001, 002, 003, 004) in the REAL-Colon (RC) dataset can be added, or all these videos can be included (001–004). For the validation set, we use the PolypGen dataset, valued for its diversity of images sourced from multiple centers and its rigorous frame-by-frame quality control, making it the best dataset to check models for generalization performance. Finally, we define three testing sets from the RC dataset **Table 4**: the first encompasses all its videos (RC); the second excludes the first five videos from each study (RC*_MT_*, REAL-Colon minus train) to assess the performance of models trained on these videos; and the third comprises only frames captured between the initial and final appearance of each polyp (RC*_SSP_*, REAL-Colon start-stop polyp). This third set is designed to mimic datasets that consist only of polyp clips, thereby discarding a large portion of the negative frames typically encountered during a full procedure. These distinct training, validation, and testing datasets provide a platform for developing polyp detection algorithms and benchmarking them in a scenario closely mimicking the actual clinical environment in which CADe systems would operate.

**Table 3:** Frame and polyp counts for training dataset splits proposed by this work including UCD and the first five videos from each clinical study in RC.

Train Sets	UCD	UCD + 001	UCD + 002	UCD + 003	UCD + 004	UCD + 001–004
**Polyps**	380	388	398	399	386	431
**Positives**	118,992	139,020	152,275	178,288	138,925	251,532
**Negatives**	217,237	383,273	355,564	565,601	347,372	1,000,099
**Frames**	336,229	522,293	507,839	743,889	486,297	1,251,631

**Table 4:** Frame and polyp counts for testing and validation dataset splits proposed by this work for deep polyp detection model evaluation.

	Test Sets			Validation Set
Set Name	RC	RC*_MT_*	RC*_SSP_*	PolypGen
**Polyps**	131	80	131	Unclear
**Positives**	342,109	209,569	342,109	3,121
**Negatives**	2,415,614	1,632,752	240,888	4,916
**Frames**	2,757,723	1,842,321	582,997	8,037

YOLO (You Only Look Once) v7 is a one-stage object detection model chosen for this study due to its exceptional speed and accuracy among other real-time object detectors (25). It uses a Cross Stage Partial Network (CSPNet), specifically CSPDarknet53, as its backbone, which is complemented by an Efficient Layer Aggregation Network (ELAN) for enhanced feature aggregation. The model incorporates a neck network based on the PANet architecture, employing a bidirectional fusion approach that integrates both top-down and bottom-up pathways for efficient feature integration. The architecture is finalized with a head network that consists of three detection heads, facilitating precise object detection across a variety of sizes (25).

All models trained in this work are YOLOv7 models targeting a single class (polyp) and were trained and tested with input images rescaled to a resolution of 640×640. Extensive data augmentation techniques were applied to enhance dataset variability and improve model generalization, in accordance with methodologies documented in related polyp detection research (26). The best data augmentation technique, adopted for all the model training in this work, employed HSV color jittering with adjustments in hue (0.1), saturation (0.5), and value (0.5), image rotation (90 degrees), translation (0.2 of image size) and flipping both vertically and horizontally, each applied with a probability of 0.5. Additionally, images were rescaled by a factor of 0.4 and mosaic data augmentation was used with a probability of 0.3 to further enhance dataset variability and model generalization. Models were trained using the SGD optimizer for 50 epochs with a batch size of 64, starting with an initial learning rate of 10^−3^. A linear scheduler was employed to adjust the learning rate to a final value of 10^−4^, with a momentum setting of 0.937. Each training epoch included all the positive frames from the training dataset, along with 50% of the total number of positive frames selected at random from the pool of negative frames. This approach was necessary to maintain a 2:1 ratio of positive to negative frames, which yielded the best results. All the models were trainined on an NVIDIA A100 GPU, with all code developed in PyTorch. At the end of the training, the model that achieved the highest mean average precision on the validation (PolypGen) set was selected for testing on RC data.

### 2.3 Per-bounding box metrics

In computer vision and medical image analysis, the evaluation of deep polyp detection models often relies on per-bounding box metrics using the Intersection over Union (IoU) criterion. A model typically outputs several bounding boxes per frame, each with a different detection score. Given a detection threshold *δ*, only predicted bounding boxes *B* with scores exceeding *δ* are considered. A bounding box *B* is classified as a true positive (*TP*) when it meets or exceeds an IoU threshold, defined as in **Equation 1** by:


(1) $$\text{IoU}\left(B,GT\right)=\frac{\left|B\cap^{\;}GT\right|}{\left|B\cup^{\;}GT\right|}$$

A (*FP*) occurs when *B* does not meet this IoU threshold, indicating an inaccurate or misplaced detection. Conversely, a false negative (*FN*) occurs when a ground truth box *GT* does not have a corresponding predicted box *B* that meets the IoU threshold. Precision (*P*) and Recall (*R*) can be computed as shown in **Equations 2** and **3**:


(2) $$P=\frac{TP}{TP+FP}$$

(3) $$R=\frac{TP}{TP+FN}$$

Detection thresholds *δ* may vary. Average Precision (AP), used to summarize and rank the performance of a model, is derived from the area under the Precision-Recall curve:


(4) $$AP=\sum_{n}(R_{n}-R_{n-1})\cdot P_{interp}\left(R_{n}\right)$$

where $R_{n}$ and $R_{n-1}$ in **Equation 4** represent consecutive recall levels, and $P_{interp}\left(R_{n}\right)$ is the interpolated precision at each recall level $R_{n}$ . AP is typically computed at a single IoU threshold, such as AP_0.5_, and averaged over IoU thresholds ranging from 0.50 to 0.95 in increments of 0.05 (AP_0.5:0.95_), to reflect various levels of detection stringency and model localization accuracy. In this work, we use AP_0.5:0.95_ as the metric for evaluating and ranking the performance of polyp detection models at a per-bounding box level.

### 2.4 Per-frame metrics

Transitioning to frame-level evaluations, a frame is classified as a TP when all polyps within the image are detected by the CADe system with an IoU exceeding a threshold, typically set at 0.2. A frame is classified as a FP when the model incorrectly predicts a polyp in a frame where none are present. Conversely, a FN occurs when a frame containing one or more polyps fails to have all polyps detected with the required IoU threshold. A true negative (TN) is defined when no polyps are present and none are incorrectly detected. The True Positive Rate (TPR) and the False Positive Rate (FPR) can be defined as in **Equations 5** and **6**.


(5) $$TPR=\frac{TP}{TP+FN}$$

(6) $$FPR=\frac{FP}{FP+TN}$$

A ROC curve, plotting TPR against FPR, varies with the detection threshold *δ*, serving as a function of the detection confidence score and aiding in evaluating model performance or selecting optimal operational parameters for the model. In this work, we use the area under the ROC curve (AUC) as the metric for evaluating and ranking the performance of polyp detection models at the frame level.

### 2.5 Per-polyp and per-patient metrics

The Per-Box Precision-Recall curve and the Per-Frame ROC, alongside metrics such as AP_0_._5:0_._95_ and ROC area under the curve (AUC), provide limited insight into a model’s clinical performance and only partially capture its practical utility. In the clinical literature on CADe systems, measures such as recall per-polyp and FP events per patient are frequently used (15, 19). However, merely defining recall per-polyp as the proportion of polyps detected in at least one frame, and FP events per-patient as the rate of FP frames per total frames, does not adequately address the key colonoscopy challenges. A more effective approach would be to assess recall at the earliest moments of detection and to identify FPs that significantly disrupt an endoscopist’s workflow. This leads to the development of metrics based on event frequency (as schematized in **Table 1**), which consider consecutive detections over a specified time length, providing a more nuanced assessment of a CADe system’s operational effectiveness.

Given a detection event length threshold, *τ*, we define a detection event as a sequence of consecutive bounding box detections spanning *τ* seconds. A FP event occurs when each bounding box within this sequence fails to sufficiently overlap with any GT bounding box, defined by an IoU of at least 0.2. We compute the average number of FP events per patient (or per full colonoscopy procedure) and denote this metric as “Average FP Events Per-Patient,” which offers a more clinically relevant assessment of FPs during a procedure. A TP event is defined when every bounding box within a detection event overlaps with a GT bounding box, achieving an IoU of 0.2 or greater within the detection window of the first *σ* seconds of a polyp’s appearance. We define the percentage of polyps that are correctly identified within *σ* seconds of their appearance as the “Per-Polyp Recall”.

A FROC curve can be constructed by graphing the per-polyp recall within *σ* seconds against the average number of FP events per patient, events defined as a function of length threshold *τ*. This is achieved by fixing two out of three parameters—detection event length threshold (*τ*), detection window (*σ*), and the polyp detection model hyperparameters (in the case of YOLOv7, *δ*)—and varying the remaining parameter. This FROC provides a visual representation of the trade-off between polyp detection effectiveness at the earliest stages of polyp appearance and the frequency of false alarms. In this work, we utilize the FROC to evaluate polyp detection models and to provide insights into each model’s clinical behavior on full-procedure videos.

## 3 Results

All code required to download, format, and prepare the five open-access datasets used in this study within the proposed platform, as well as to generate the ROC and FROC plots reported in this section, is fully available at https://github.com/cosmoimd/polyp_detection_platform. Additionally, the code to reproduce all YOLOv7 experiments described in this section is available at https://github.com/cosmoimd/yolov7.

### 3.1 Per-bounding box results

In **Table 5**, we present the results on RC*_MT_* dataset in terms of AP_0_._5:0_._95_ for the six models trained in this study. The results indicate that the four RC dataset cohorts vary significantly in terms of difficulty, with cohort 003 proving to be the most challenging. When analyzing the test results, we divided RC*_MT_* into four subsets, each corresponding to a different cohort. As anticipated, models trained on data specific to a particular cohort generally performed better on test data from that same cohort, with the model trained on the largest dataset yielding the best results.

**Table 5:** Average Precision (AP_0_._5:0_._95_) results on RC*_MT_* and its splits corresponding to one of the four cohorts.

Train Sets	Test Sets				
	001	002	003	004	001–004
UCD	0.267	0.242	0.171	0.269	0.229
UCD + 001	0.323	0.261	0.192	0.230	0.239
UCD + 002	0.292	0.289	0.201	0.265	0.253
UCD + 003	0.284	0.265	0.228	0.285	0.260
UCD + 004	0.273	0.258	0.187	0.301	0.247
UCD + 001–004	**0.377**	**0.359**	**0.275**	**0.343**	**0.331**

Models trained with data from specific cohorts showed improved performance on test sets from those same cohorts (underlined results). Training with data from all centers resulted in the best overall performance (bolded).

For the model trained solely on the UCD dataset, we report the test results on the entire RC dataset in **Table 6**. The model achieves a precision of 0.51 and a recall of 0.38 per-box at its optimal working point of *δ* = 0.6 (the point achieving the best F1 score). We also present results for this model under different formulations of the RC dataset: RC*_SSP_* and two subsets of RC, one containing only 100 positive frames per polyp without negatives (RC_100,0_), and another with 100 positive frames per polyp and 10% random negatives RC_100,0.1_. Additionally, we categorize results based on box sizes—small (less than 100 px), medium (less than 200 px), and large (the rest)—as defined in previous research (24). It is notable that the model struggles with small bounding boxes and how different test set formulation provide different results.

**Table 6:** Average precision (AP) metrics for a YOLOv7 model trained on the UCD dataset, tested across different picks of the complete RC dataset.

Metric	Polyp Size	RC	RC_100,0_	RC_100,0.1_	RC*_SSP_*
AP_0.5:0.95_	all	0.24	0.325	0.163	0.315
AP_0.5_	all	0.372	0.539	0.253	0.507
AP_0.50:0.95_	small	0.017	0.046	0.014	0.035
AP_0.50:0.95_	medium	0.163	0.262	0.125	0.231
AP_0.50:0.95_	large	0.333	0.482	0.265	0.42

### 3.2 Per-frame results

**Figure 1:**
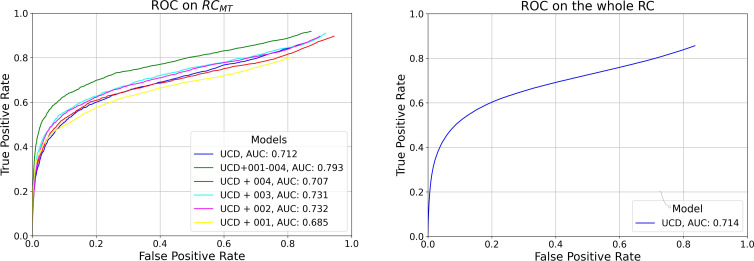
Left: Per-Frame ROC curves for the six models trained in this study, evaluated on RC*_MT_*. Right: Per-Frame ROC for the model trained exclusively on the UCD dataset, tested on the entire RC dataset.

In **Figure 1**, we present ROC curves for the six models trained in this study tested on RC*_MT_* varying YOLOv7 model confidence score threshold *δ*, along with the AUC for each model. The trend observed aligns with the findings from the AP_0.5:0.95_ evaluation, indicating that the model trained on the most comprehensive dataset, which includes all RC study data (UCD+001–004) and thus uses the largest volume of data, achieves the best performance. For the model trained exclusively on the UCD dataset, we report a True Positive Rate (TPR) and FP Rate (FPR) of 0.40 and 0.04 per-frame on the whole RC dataset, respectively, at its optimal working point (0.6, selected during the per-box evaluation phase).

### 3.3 Per-polyp and per-patient results

**Figure 2:**
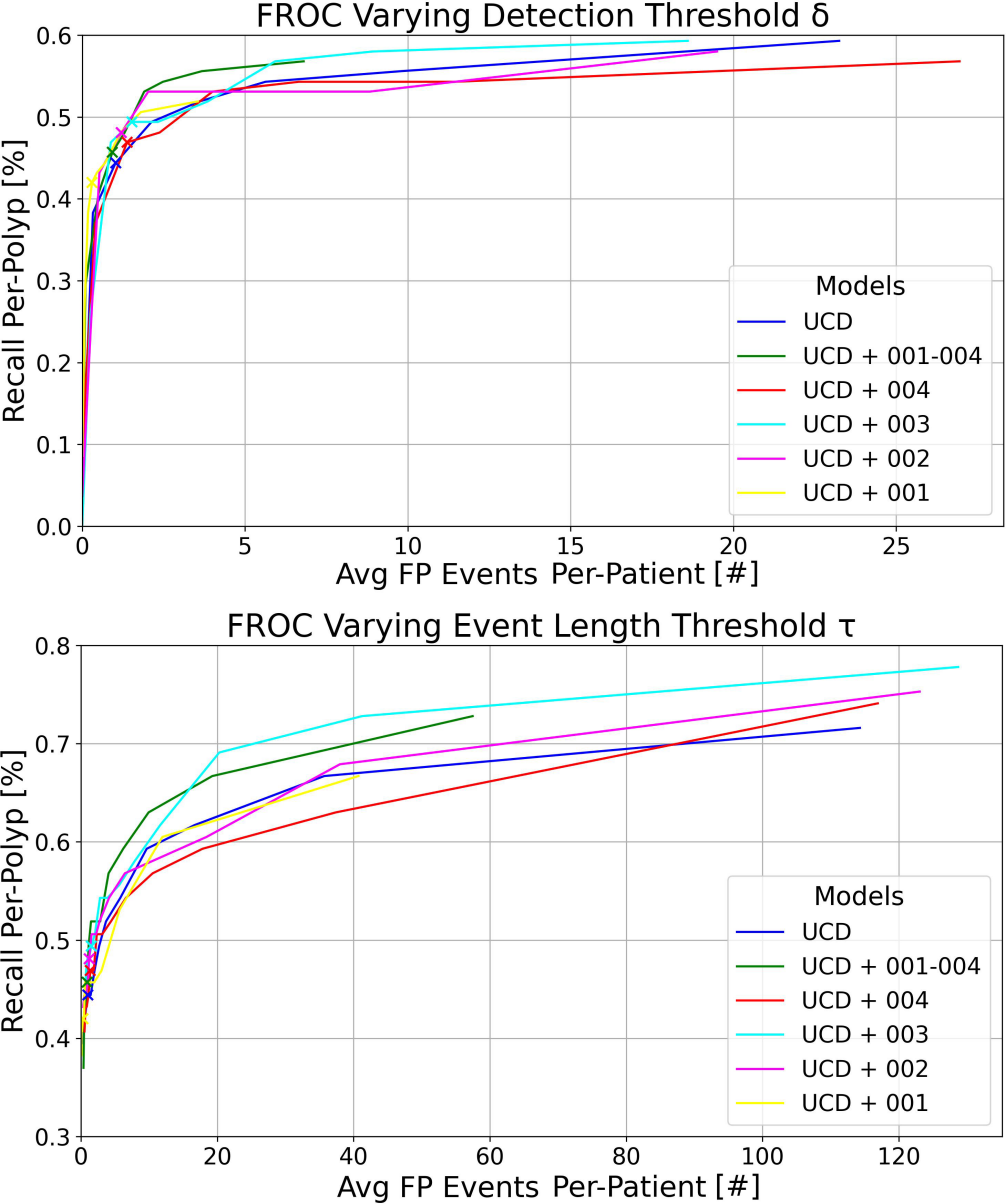
Top: FROC plots illustrating the variation in detection threshold *δ* for the six models trained in this study, evaluated on RC*_MT_* with a detection event length threshold *τ* = 2*s*. Bottom: FROC plots showing the impact of different event length thresholds *τ* for the same models on RC*_MT_*, with a fixed detection threshold *δ* = 0.6. In both plots, the detection window is set at *σ* = 3*s* and the IoU threshold at 0.2.

In **Figure 2**, we display the FROC curves for the six models evaluated on RC*_MT_*. The top section of the figure shows curves calculated at various confidence score thresholds (*δ*) for the YOLOv7 models, with a detection event length *τ* = 2*s* and a detection window *σ* = 3*s*. Each point on these curves represents the percentage of polyps detected within 3 seconds of their appearance by an event where consecutive bounding box predictions span 2 seconds, and at least one detection overlaps with at least one ground truth (GT) polyp bounding box by an IoU of 0.2. The confidence score threshold *δ* ranges from 0.1 to 0.9, with the point at 0.1 positioned in the top right of the plot, indicating an average of more than 20 FP events per patient and a per-polyp recall greater than 50%. Increasing *δ* leads to a decrease in both the number of FP events per patient and the recall per-polyp. The x-marked points correspond to *δ* = 0.6 - the optimal model working point as determined in the per-box analysis for maximizing the F1 score.

The bottom plot of **Figure 2** illustrates the curves calculated at various *τ* thresholds for the YOLOv7 models, maintaining a detection window of *σ* = 3*s*. This analysis was performed by setting the confidence score *δ* at 0.6 and varying *τ* from 0.2 (resulting in the top right of the plot with a high average number of FP events per patient and high recall per-polyp) to 3, in increments of 0.2. The x-marked points correspond to *τ* = 1*s*.

**Figure 3:**
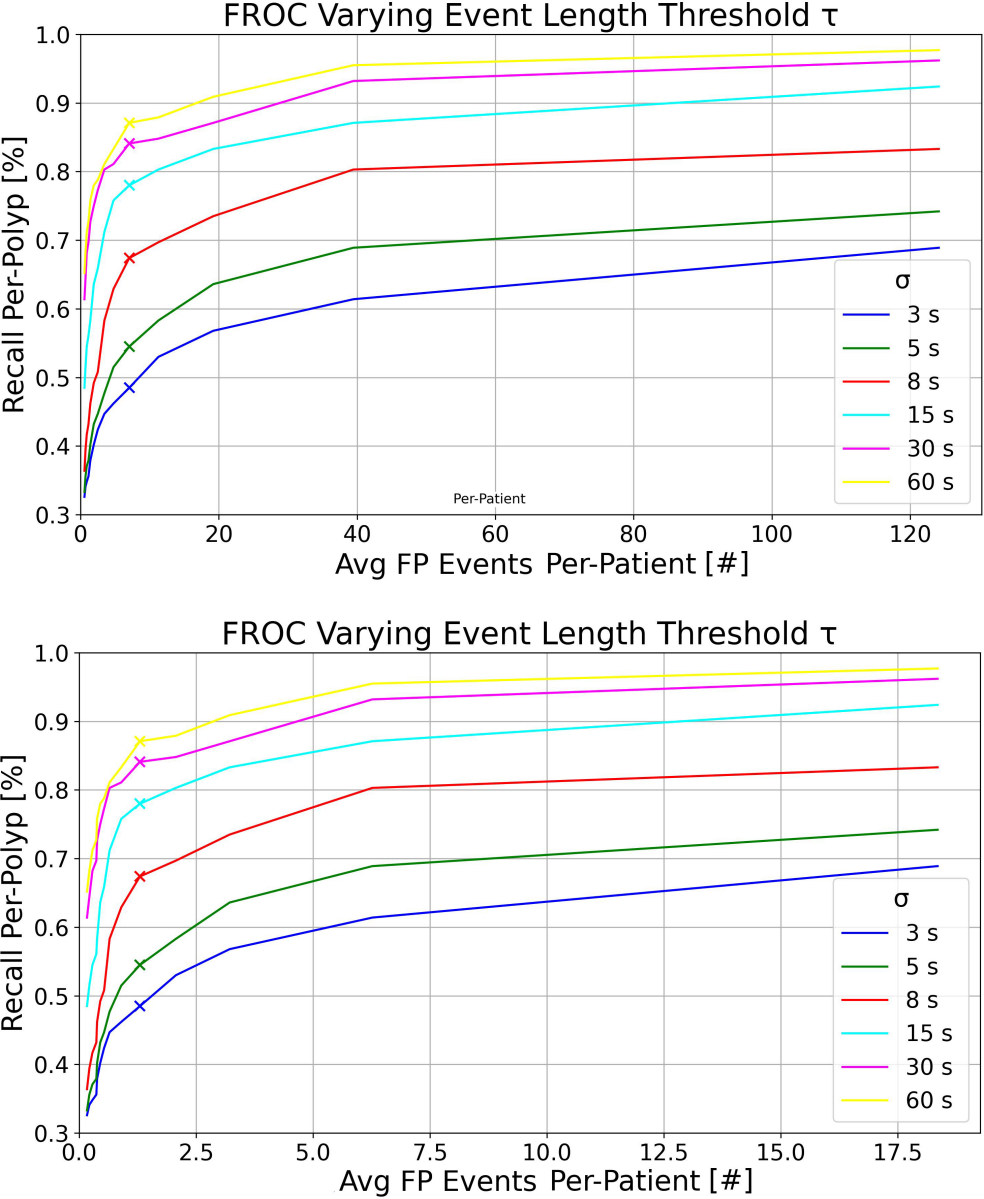
Top: FROC plots for the UCD model tested on the RC dataset, varying the detection event length threshold *τ* for multiple detection windows *σ*. This was executed with detection threshold *δ* = 0.6 and IoU threshold = 0.2. Bottom: FROC plots for the UCD model tested on the RC*_SSP_* dataset, using the same parameters as above.

At the top of **Figure 3**, we present the FROC curves for the model trained exclusively on the UCD dataset, tested on the whole RC dataset. Each curve represents different detection event length thresholds *σ* on the complete RC dataset, with *τ* varying from 0.2 to 3 seconds and *δ* set at 0.6. It is evident that increasing *σ*, i.e., the maximum detection time window in which an event can capture at least one GT polyp bounding box from its first appearance, leads to higher recall per-polyp rates. At the bottom of **Figure 3**, we display the same curves computed on the RC*_SSP_* data split, which only considers frames between the first and last appearances of each polyp. Although the trends in RC*_SSP_* are similar, there are significantly fewer FP events per patient. This reduction is attributed to the model being tested on a significantly larger number of negative frames, as detailed in **Table 4**.

Overall, as observed in the bottom plot of **Figure 3**, the new metrics preserve similar model ranking as seen in the per-frame ROC plot of **Figure 1** and in **Table 5** in the per-box analysis. However, the results become more clinically interpretable, incorporating a definition of FPs that more effectively describes both the disturbance to the endoscopist and performance at early polyp discovery. For instance, the top plot of **Figure 3** shows that with a detection event formation threshold (*τ*) of 1 second, the model trained solely on the UCD dataset achieves a per-polyp recall of 48.5% within a response time (*σ*) of 3 seconds. This indicates that the off-the-shelf YOLOv7 model, when trained on UCD data, can detect half of the polyps in the REAL-Colon dataset within 3 seconds, generating seven FP events per patient. Extending the response time to *σ* of 15 seconds increases the per-polyp recall to 78%, without affecting the average number of FPs. Using the same detection threshold (*δ*) of 0.6 previously resulted in a per-box precision of 0.51 and recall of 0.38 (**Table 5**), and a per-frame TPR of 0.4 and FPR of 0.04 (**Figure 1**, right), which are less interpretable for clinical use.

## 4 Discussion

In this study, we demonstrate how the recent release of full-procedure, unaltered colonoscopy videos from the REAL-Colon dataset, along with other open-access colonoscopy datasets, facilitates comprehensive research, development, and clinic-like evaluation of learning-based models for polyp detection in a fully open-access environment. To this end, we have developed an open-access platform that integrates four open-access datasets for model training and validation, using the REAL-Colon dataset as a fifth dataset for testing. This platform offers data splits for consistent model development and benchmarking, and introduces new application-specific metrics. These metrics, accompanied by corresponding FROC plots, are tailored to meet clinical needs more closely than standard object detection metrics. Specifically, they focus on events of consecutive detections over a specified time length *τ* and include per-polyp recall within *σ* seconds and FP event rates per patient.

By setting a detection event formation threshold of *τ* = 1*s* and assessing per-polyp recall within *σ* = 3*s* of polyp appearance, the model trained on the UCD dataset achieved a per-polyp recall of 48.5% on the REAL-Colon dataset, while generating seven FP events per patient at the optimal working point *δ* = 0.6. *δ* is usually set to maximize the F1 score (as in the case of this work) or other per-box metrics, but it can now be adjusted to better meet clinical needs by quantifying FP events at a patient/procedure level and measuring early polyp detection. Reports from commercially available CADe systems suggest that an FP rate of two or fewer per patient is typical for events lasting one to two seconds Cherubini and Dinh (15)Hassan et al. (19). Consequently, a rate of seven FPs per patient might be considered excessively high; therefore, increasing *δ* to lower this rate would make the CADe system less intrusive (even if reducing per-polyp recall). Such adjustments not only enhance CADe behavior interpretation in clinical practice but also enable the effective fine-tuning of system hyperparameters by providing complementary information to traditional metrics. Furthermore, they could facilitate model comparisons at identical clinical working points (such as a given value of FP per-patient).

As demonstrated in **Table 6**, our findings also emphasize the importance of using the complete set of video frames from full procedures in the test set of our proposed platform, rather than a subsample, to achieve a more accurate assessment of FP events at the patient level. Analyzing only frames from polyp clips, as shown in **Figure 3** (bottom), results in a nearly eightfold underestimation of FP events per patient, primarily due to the exclusion of a large number of negative frames, which make up nearly 90% of a typical colonoscopy video (16, 17). Moreover, using continuous frames without gaps is essential for accurately computing per-polyp recall within *σ* seconds, which would not be possible otherwise. For these reasons, we chose the REAL-Colon dataset as the testing set for our open-access platform. Additionally, this dataset enables the derivation of metrics across various bounding-box sizes of the same polyps. The results underscore that detecting smaller bounding boxes presents the greatest challenge but it is crucial for effectively identifying hard-to-spot polyps, corroborating findings from previous research (27).

Another noteworthy feature of the proposed open-access platform is its capacity to assess models performance using data from multiple centers. In the proposed platform, model checkpoints were selected based on their maximization of AP_0.5:0.95_ on the PolypGen dataset, chosen for its diverse representation of polyps from various centers. The UCD training dataset comprises over 118,000 positive images, including 380 polyps, and over 217,000 negative images sourced from multiple sites, distinct from the validation and test sets. This extensive dataset forms a solid foundation for model training. Additionally, the REAL-Colon test set encompasses video data from four diverse cohorts collected worldwide, facilitating the evaluation of model generalization across four unseen centers (United States, Italy, Austria, Japan). Taken together, this platform enables comparison of various models developed and validated under the same conditions, thereby ensuring the robustness and reliability of CADe system development. As anticipated, our results demonstrate that models trained on specific cohorts exhibited enhanced performance on their corresponding test sets, with an average 5-point increase in average precision achieved by incorporating just 5 videos from the same cohort in the training set (**Table 4**). An important source of error arises from the variability in endoscope models and types of illumination used across different data cohorts, and fine-tuning on center-specific data helps address this issue. Notably, cohort 003, characterized by the lowest average Boston Bowel Preparation Scale (BBPS) score of 7.8 compared to scores of 8.3 or higher in the other cohorts and featuring the longest video acquisitions, resulted in the lowest AP_0.5:0.95_. This is likely due to a higher number of frames containing debris that resembles polyp-like structures and can confound the model or that complicate the detection of actual polyps. We believe that future efforts should prioritize the development of models with robust generalization capabilities across all four cohorts.

Finally, the importance of real-time processing in deploying polyp detection models in clinical environments is crucial. It ensures the continuity of clinical procedures without introducing disruptive delays. All YOLOv7 models trained in this study achieve an inference speed of 85 frames per second with a batch size of one on an NVIDIA RTX A4000 with 16GB, aligning well with the typical endoscopy video frame rate of 25–30 frames per second. Moreover, potential enhancements using acceleration libraries such as TensorRT could further optimize this efficiency. The ultimate objective of polyp detection research is to develop models that can achieve real-time processing speeds, maintain a minimal number of FP events per-procedure, and maximize per-polyp recall within *σ* seconds. Our proposed platform facilitates the realization of these goals.

A limitation of this study is the exclusive use of the off-the-shelf YOLOv7 model, which, while demonstrating adequate sensitivity and specificity for polyp detection, primarily serves as a baseline. This approach was intentional, aimed at establishing and validating new clinical metrics and showcasing the proposed open-access platform. We have made the YOLOv7 code and processing scripts fully available to enable easy replication of our experiments and to encourage the exploration of alternative models in future research.

In conclusion, this research introduced a novel open-access platform that enhances the development and evaluation of deep polyp detection models under clinically equivalent conditions. The platform includes rich training and validation datasets and a testing dataset comprised of full-procedure videos, enabling clinic-like validation and model generalization assessment across unseen centers, particularly in challenging situations such as early detection and small polyp bounding box sizes. Moreover, we introduced new metrics and corresponding FROC curves designed to more effectively interpret the efficacy of polyp detection algorithms and fine-tune their working hyperparameters. Combined with estimation of real-time processing capabilities, these tools facilitate a comprehensive evaluation of a model’s clinical efficacy. By leveraging these innovations, we hope that future research will significantly enhance patient care by better meeting the clinical demands of real-time CADe colonoscopy.

## Data availability statement

The original contributions presented in the study are included in the article/supplementary material. Further inquiries can be directed to the corresponding author.

## Author contributions

GM: Data curation, Methodology, Software, Writing – original draft, Writing – review & editing, Formal analysis, Visualization. PS: Data curation, Formal analysis, Methodology, Writing – original draft, Writing – review & editing. AC: Formal analysis, Methodology, Writing – original draft, Writing – review & editing, Conceptualization, Project administration. CB: Conceptualization, Formal analysis, Methodology, Project administration, Writing – original draft, Writing – review & editing, Data curation, Software, Supervision, Visualization.
